# Treatment-Free Remission—A New Aim in the Treatment of Chronic Myeloid Leukemia

**DOI:** 10.3390/jpm11080697

**Published:** 2021-07-22

**Authors:** Paulina Kwaśnik, Krzysztof Giannopoulos

**Affiliations:** 1 Department of Experimental Hematooncology, Medical University of Lublin, 20-093 Lublin, Poland; krzysztof.giannopoulos@gmail.com; 2 Department of Hematology, St John’s Cancer Center, 20-090 Lublin, Poland

**Keywords:** chronic myeloid leukemia (CML), tyrosine kinases inhibitors (TKIs), treatment-free remission (TFR), TKI withdrawal syndrome

## Abstract

Tyrosine kinases inhibitors (TKIs) revolutionized chronic myeloid leukemia (CML) treatment for many years, prolonging patients’ life expectancy to be comparable to age-matched healthy individuals. According to the latest the European LeukemiaNet (ELN) recommendations, CML treatment aims to achieve long-term remission without treatment (TFR), which is feasible in more than 40% of patients. Nearly all molecular relapses occur during the first 6 months after TKI withdrawal and do not progress to clinical relapse. The mechanisms that are responsible for CML relapses remain unexplained. It is suggested that maintaining TFR is not directly related to the total disposing of the gene transcript *BCR-ABL1*, but it might be a result of the restoration of the immune surveillance in CML. The importance of the involvement of immunocompetent cells in the period of TKI withdrawal is also emphasized by the presence of specific symptoms in some patients with “withdrawal syndrome”. The goal of this review is to analyze data from studies regarding TFRs in order to characterize the elements of the immune system of patients that might prevent CML molecular relapse. The role of modern droplet digital polymerase chain reaction (ddPCR) and next-generation sequencing (NGS) in better identification of low levels of BCR-ABL1 transcripts was also taken into consideration for refining the eligibility criteria to stop TKI therapy.

## 1. Introduction

Chronic myeloid leukemia (CML) is the first malignant monoclonal disease of hematopoietic stem cells (HSCs) with identification of an acquired chromosomal abnormality: the Philadelphia chromosome (Ph) [1]. This chromosome 22 is the result of a balanced exchange of genetic material between the long arms of chromosome 9 and 22 (translocation, t(9;22)(q34;q11)), generating juxtaposition of the *BCR* gene in 9q to the *ABL1* proto-oncogene in 22q [2,3]. Expression of this fusion gene produces the BCR-ABL1 oncoprotein whose tyrosine kinase activity deregulates many signal transduction pathways in the HSC. The BCR-ABL tyrosine kinase activity represents the primary alteration in CML, proved in vitro as well as in animal models. The oncogenic BCR-ABL1 protein (P210^BCRABL1^) is responsible for the phosphorylation of tyrosine residues on individual substrates, such as adapter, catalytic, cytoskeleton, and membrane proteins. Moreover, due to autophosphorylation, binding sites are created for the SH2 domains of other proteins by increasing phosphotyrosine on BCR-ABL1. Malignant transformation mainly concerns mechanisms related to altered adhesive properties, activation of mitogenic signaling pathways, inhibition of apoptosis, and degradation of inhibitory proteins. The inhibition of the adhesion of CML progenitor cells to the bone marrow stromal cells and the extracellular matrix is mainly related to the influence of BCR-ABL1 on integrin dysfunction. Mitogenic signaling is important for the pathogenesis of Ph-positive cells. BCR-ABL1 protein has been shown to be associated with disturbances in Ras mitogen-activated protein kinase (MAPK), leading to increased proliferation. The Jak-Stat pathway, and more precisely, constitutive phosphorylation of the transcription factors Stat1 and Stat5, lead to the impairment of transcriptional activity and promote the anti-apoptotic effect. The phosphoinositide 3-kinase (PI3K) induces the proliferation of leukemic cells, thereby inhibiting apoptosis, which may also be affected by Myc overexpression. Maliciously transformed BCR-AB1 cells prevent apoptosis by blocking the activation of caspases and thanks to the aforementioned Ras protein or PI3 kinase [4]. The natural evolution of CML consists of three phases: a chronic phase (CP-CML) that without tyrosine kinases inhibitor (TKI) therapy could last 3–4 years with harmonic malignant HSC differentiation; an accelerated phase (AP-CML) with HSC maturation hiatus and a blastic phase (BP-CML) in acute leukemia—myeloblastic or lymphoblastic—with a fatal outcome within a few months. This three-phase evolution has been modified by the clinical results of TKIs, and since then CML is no longer a fatal disease but rather a chronic condition with age-related diseases. TKIs revolutionized CML treatment for many years, and prolong patients’ life expectancy to be comparable to age-matched healthy individuals [5,6,7,8,9]. With significant advances in the treatment of CML, priority is placed on improving the quality of life of CML patients and on the efficacy, tolerability, and toxicity of TKIs, as well as on the pharmacoeconomics of long-term treatment. Therefore, the ability to discontinue TKI treatment and achieve long-term remission without treatment (treatment-free remission—TFR) has become a new goal in CML, and the selection of appropriate therapy is a key element in the depth and speed of achieving a very deep molecular response.

## 2. Treatment

Symptomatic patients with leukocytosis and thrombocytosis may receive a dose of hydroxyurea before confirming a diagnosis of CML for a short time. Pregnancy is an absolute contraindication to the treatment of TKI, then interferon alpha (IFN-α) therapy is recommended. Currently, the advent of pegylated interferon (PEG-IFN) has appeared, which is more effective and better tolerated. Recent studies have shown that PEG-IFNα in combination with imatinib can accelerate the speed and depth of molecular responses [10], but this requires further research similarly to the combinations with the second-generation (2G) TKIs with PEG-IFNα, which could also generate benefits [11,12]. Five drugs are approved by the Food and Drug Administration (FDA) and the European Medicines Agency (EMA) for the treatment of CML: imatinib; second-generation TKIs, nilotinib, dasatinib, bosutinib; and third-generation TKI, ponatinib. Rodotinib, another 2G TKI, has only been approved in South Korea. Generic imatinibs are alternative drugs for the first-line treatment, which are already widely available and became affordable with significantly lower costs than the brand product [13,14,15,16].

The general principle of operation of most used TKIs is to inhibit the activity of ABL1 tyrosine kinase in an ATP-competitive manner with different specificities. Treatment of BCR-ABL1 transformed cells inhibits their proliferation and induces apoptosis. However, it has been proven that despite maintaining a stable deep molecular response (DMR), leukemic cells can still be detectable but might be under the control of the immune system [17]. Imatinib binds to the ABL1 kinase domain when it has a catalytically inactive conformation. The mutations in the binding site of imatinib (within the SH2 contact, P loop positions: M244, G250, Q252, Y253, E255, T315, F317, M351, F359, H396) cause resistance to this drug, e.g., promoting the active conformation of the enzyme [18]. Nilotinib, despite its increased affinity, binds to the same pocket as imatinib, therefore therapy with this drug is also associated decreased activity against mutations in positions: Y253, E255, T315, F359, but it is effective against some mutations resistant to imatinib [19]. In parallel with the advancement of nilotinib, a dual SRC/ABL inhibitor dasatinib was developed, which binds to the active conformation of the ABL1 kinase, showing even higher affinity than previous drugs. The dasatinib resistance mutations are V299, T315, and F317 [20]. A similar binding mechanism to dasatinib is demonstrated by bosutinib, the other SRC/ABL inhibitor with mutations resistant to this drug similar to that of dasatinib, and additionally G250 and E255. Table 1 summarizes the first- and second-generation TKIs in the treatment of CML.

Ponatinib is 3G TKI drug, potent inhibitor of ABL1 kinase, and is effective against the described resistance mutations to other TKIs, including T315I. The T315I mutation, which is a point mutation, and due to its location at the entrance to the ATP binding site determines resistance to imatinib, nilotinib, dasatinib, and bosutinib. In different trials, ponatinib showed inhibitory activity against native BCR-ABL1 kinase and several ABL1 mutations. For this reason, ponatinib is currently indicated for the treatment of CML in every phase of the disease. The risk of resistance to ponatinib may be due to several general drug-related mechanisms, such as inhibition of single or multiple non-therapeutic target kinases, possible drug–drug interactions, or direct pharmacological toxicity, but may be due to the occurrence of still unknown mutations. However, due to the broad target profile of ponatinib, it has a high risk of vascular occlusive events, which has limited its potential in the treatment of CML in the first line but represents the drug of choice for T315I mutants as well as in subsequent therapy lines [30]. The T315I point mutation has long been a clinical challenge, so a new promising drug, asciminib, which was first approved by the FDA in February 2021, is an attractive approach to the treatment of CML, especially for patients with the T315I mutation. Asciminib is a STAMP (specifically targeting the ABL myristoyl pocket) inhibitor that does not bind to the ATP binding pocket and thus exhibits a mechanism of action different from that of commonly used TKIs. Asciminib as an allosteric ABL1 kinase inhibitor stabilizes the inactive conformation of the enzyme by binding to the ABL1 myristoyl pocket. The encouraging results of asciminib have shown its safe and clinically effective profile and, moreover, it might be a therapeutic option in patients with T315I, including those with ponatinib resistance/intolerance [31]. Another new substance—olverembatinib, known as HQP1351—also proved effective in the II phase of clinical trials in CML patients with a broad spectrum of mutations, including the T315I mutation [32]. Vodobatinib was studied in a phase I clinical trial in a group of CML patients who failed more than three different TKIs divided into groups not treated with ponatinib as well as those treated. Thanks to the comparative effectiveness in both groups, it may become a promising agent for the treatment of resistant CML [33]. When resistance occurs on the first-line drug, it is obligatorily changed to another TKI. Each change to a different TKI is undertaken by the existing mutations, age, comorbidities, and individual preferences of the patient [34]. In particular, the risk of cardiovascular complications should be taken into account when ponatinib is started [35,36]. The PACE study showed that ponatinib provided sustained and clinically meaningful responses that were sustained even after dose reduction [37], but the incidence of arterial occlusive events (AOEs) is relatively high [35].

The most mature data for TFR and the availability of the generic version supports the primary utilization of imatinib for initial TFR attempts. In fact, the use of 2G TKIs as first-line treatment leads to faster molecular responses [38] and patients treated with nilotinib or dasatinib in the first line develop fewer resistance mutations to other TKIs and therefore can be eligible for TFR faster, as demonstrated in the DASSION study [21] and ENESTnd [22]. However, there are some concerns regarding the toxicity of 2G TKIs, which are more toxic then imatinib. In more than 50% of medium- and high-risk patients, as assessed by the new EUTOS long-term survival (ELTS) score, the benefits of 2G TKIs were reported in terms of extended overall survival (OS). The BFORE study showed that bosutinib used as subsequent line treatment in patients with resistance or intolerance to prior therapies was more effective at generating faster and deeper molecular responses than imatinib, but more frequent adverse events, such as diarrhea and elevated levels of aminotransferases, were reported [28]. Furthermore, 2G TKIs are also very effective in patients diagnosed with CML in the accelerated phase. A French study reported that after 7 years of follow-up, the OS in these patients was 87% and the PFS was 83%.

## 3. AlloSCT

Until now, the only potential cure for CML has been allogeneic stem cell transplantation (alloSCT). However, nowadays, alloSCT is a suitable option only for a limited group of patients due to the effectiveness of TKIs. The lack of a sustained response to 2G TKIs and ponatinib suggests a high risk of disease progression, which is an indication for an alloSCT. Patients with AP-CML or progressing to AP-CML also qualify for alloSCT due to the high risk of disease progression. In BP-CML, alloSCT is not recommended, thus every effort should be made to achieve a second CP-CML and then perform an alloSCT immediately [39,40,41].

## 4. Monitoring and Prognostic Factors

Due to the detection of additional aberrations, classical cytogenetics is of importance, especially at the time of diagnosis. Cytogenetics testing should be performed in patients with atypical translocations or *BCR-ABL1* transcripts, treatment resistance to exclude additional chromosomal aberrations (ACA), and with progression to AP-CML/BP-CML. The FISH method may also be helpful in detecting abnormal transcripts. Although it is a good quantitative tool, it is currently not recommended for use when considering TFR [42].

The molecular response (MR) is the ratio of *BCR-ABL1* transcripts to *ABL1* transcripts or to other control transcripts according to the international scale (IS) and is expressed as *BCR-ABL1*% on a log scale according to the IRIS study (*BCR-ABL1* 1% correspond to a decrease of 2 logs, respectively) [43,44]. The goal for the first year of therapy is to achieve major molecular remission (MMR). Soverini et al. emphasize changing the common control gene *ABL1* to *GUSB* in the first months of treatment, due to the underestimation of the actual *BCR-ABL1/ABL1* ratio in the initial stages of therapy, which is crucial. This is due to the fact that the PCR primers used for *ABL1* amplification also amplify the target sequence from the *BCR-ABL1* fusion transcript, while the use of *GUSB* as a control gene or performing a parallel assessment of *ABL1* and *GUSB* could improve the reliability of the results [18,45,46].

The Spanish TKI discontinuation report confirms the existence of two prognostic factors: length of TKI treatment, which should be at least 5 years (TFR: 59% with MR4.5 > 5 years vs. 30% with MR4.5 < 5 years), and duration of deep molecular response (MR4.5) for at least 4 years (TFR: 47% with MR4.5 > 4 years vs. 25% with MR4.5 < 4 years).

So far, to determine the prognosis of the disease in CML patients, there have been three systems—Sokal, Euro, and EUTOS. However, the ELTS score was created, which captures the same prognostic factors as the Sokal score, such as basic morphological data, spleen size, and age, but the age value has a lower negative prognostic value than the Sokal score and is important in predicting the likelihood of death related to leukemia [47,48].

The latest recommendations the European LeukemiaNet (ELN) prefer the use of ELTS because it delimits the risk groups in patients much better than the Sokal score and about 50% of patients can change allocations based on the ELTS score [49]. In the group of patients over 65 years of age, it turned out that the ELTS score identifies a group of patients who have a greater chance of achieving MMR or MR4. It is recommended for risk assessment in this group of CML patients, where it may be important in deciding to start 2G TKIs treatment as soon as possible, and even from the very beginning. Additionally, it identifies a group of patients who do not require such treatment, especially when patients are at risk of additional age-related comorbidities.

## 5. The Emerging Role of New Methods of Molecular Testing in CML-NGS and ddPCR 

Next-generation sequencing (NGS) and digital droplet PCR (ddPCR) are molecular techniques that are increasingly being considered in terms of mutation detection in CML patients. Sanger sequencing (SS) is still the gold standard in screening tests for the detection of *BCR-ABL1* kinase domain (KD) mutations, but recent studies on NGS have shown its importance especially in the detection of low-level mutations. The NEXT-in-CML study showed that mutations undetectable by SS occurred in 34% of patients and 18% (of all) had low-level mutations [50]. Due to the kinetics of TKI, resistant low-level mutations invariably expand if the patients are not switched to another TKI or appropriate TKI dose, which may be important in clinical decision-making and thus NGS should enter clinical practice. Despite the effectiveness, NGS remains an expensive and technically demanding method. In recent years ddPCR has been gaining popularity due to the linearity of the results, their accuracy, repeatability, and standardization. The greatest advantage of ddPCR is a sensitivity of around 10^7^, which is definitely beyond the limits of RQPCR [42]. A good correlation was observed between the results of ddPCR and NGS in detecting resistance mutations [51].

## 6. Treatment-Free Remission (TFR)

The clinical course of CML has changed significantly in recent years [52]. CML is identified with a chronic disease in which the effectiveness of targeted therapy translates into a much better prognosis of patients who live like healthy people [5,8,9]. Moreover, patients with CML are dying not directly as a consequence of this disease, but due to age-related comorbidities [49]. The concept of an “operational cure” emerged in connection with the increasing questions about improving the quality of life of CML patients, preventing the toxicity of long-term drug intake, and alleviating their side effects. It was based on keeping the patient in remission despite the detection of leukemic cells in minimal residual disease, which may be related to the immunological control and the type of *BCR-ABL1* transcript [53,54]. Discontinuation of TKI treatment and the achievement of sustained TFR is possible in patients who have achieved a stable deep molecular response (DMR). A DMR is understood as *BCR-ABL1* transcript levels of molecular response MR 4, MR 4.5, and MR5 on the IS (Table 2) [44,55].

**Figure 1:**
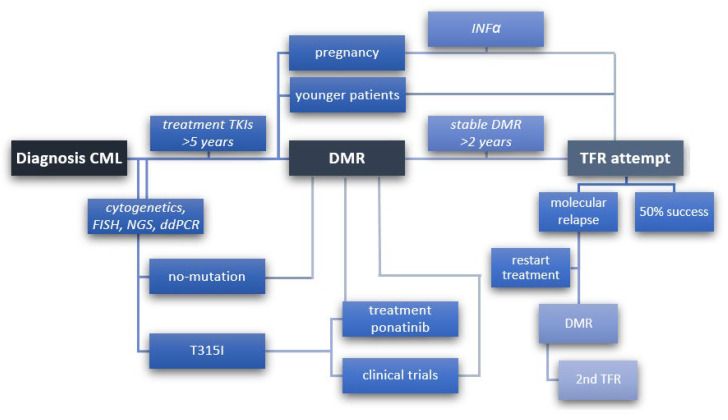
Steps to follow to achieve TFR from diagnosis of CML, detection of individual mutations, treatment of TKI to stable deep molecular remission (DMR), and then discontinuation of treatment (TFR) with 50% success or molecular relapse, prompting re-initiation of treatment to re-attain DMR and eventually make a second attempt to discontinuation treatment (2nd TFR). A scheme to include younger patients with low- to medium-risk disease and women who wish to become pregnant for whom TFR has a high priority.

In the consideration of the latest recommendations of ELN of March 2020, the aim of CML treatment is “normal survival and good quality of life without life-long treatment” [49]. Consequently, it is important to standardize the recommendations and criteria for qualifying patients for an effective TFR. In the new approach, it is possible to maintain a stable DMR without continuing treatment with TKI (Figure 1). Over time, successive attempts have been made to maintain TFR, but most are still limited to clinical trials [56].

An important issue in favor of undertaking TFR studies is the toxicity of TKIs use, which may last for many years due to the prolonged exposure of the drug to the organism. The reported adverse events may be hematological, including neutropenia, thrombocytopenia, and anemia. They are not the basis for changing the treatment and very rarely cause complications, as often the stabilization of hematological parameters requires time for the organism to adjust to the drug. Non-hematological adverse events are those that worsen the patient’s quality of life and complications that require a change in treatment due to the risk of deterioration of health or even death [49].

The implementation of TFR is justified, especially in patients who wish to become pregnant. Pregnancy in patients with CML precludes continuing TKI treatment due to their teratogenic effect [57]. It is probably related to the inhibition of organogenesis by blocking the PDGFR receptor by TKIs [49]. Women who cannot undergo TFR due to a lack of a sustained DMR can replace TKIs with IFN-α therapy.

Children are another group, apart from pregnant women, for whom a successful TFR might be of great prominence. Although the CML is rarely diagnosed in children, it is speculated that treatment may have a negative impact on their growth [58,59,60]. Currently, TFR is not attempted in pediatric patients because the clinical course of the disease is usually more aggressive than in adults [61]. However, studies in this group of patients are necessary due to exposure to the long-lasting toxicity of TKIs according to the principle of lifelong treatment.

The prevalence CML increases with better survival rates of CML patients and more than 400,000 CML patients are expected in Europe by 2050 [56,62]. The cost of treating one patient is currently EUR 30–40,000 per year in most European countries, and although these costs are expected to fall due to the availability of generic imatinib and soon also generic dasatinib, it is still a great impact on the healthcare budget [15,63].

Table 3 summarizes the results of TFR trials. TFR is in the range of 30–70%, and its rate decreases with time after treatment discontinuation in subsequent attempts. Molecular relapse has been defined in most studies as a loss of MMR, followed by treatment resumption [64]. Overall, the median molecular relapses based on the above studies is 47%, with a similar relapse rate in the discontinuation studies with both imatinib and nilotinib or dasatinib. Thus, the probability of maintaining TFR seems to be similar for both imatinib and 2G TKIs nilotinib or dasatinib discontinuation and is approximately 50%. Eighty percent of all molecular relapses occur within the first 6–8 months after TKI discontinuation [65]. TFR studies do not envisage the participation of patients treated with TKIs 3G, but such studies have been conducted. Six patients from the PAECE study entered the TFR trial, four of which are still in remission, and two experienced molecular relapse but regained DMR after re-initiation of low-dose ponatinib treatment [66].

The second stop attempt has a significantly lower rate of successful TFR. The median molecular relapse in the group of patients undergoing 2nd TFR in the above studies reached 74%. Second attempts to stop TKI in MR4.5 were successful in only 1/3 of eligible patients. The latest reports confirmed that TFR2 trials are safe and that discontinuation of treatment may be effective, but require close monitoring of the patient in an extended stage. Decisive for the success of TFR2 is the duration of the TFR for the first attempt and thus the rate of molecular relapse and the TKI-free time after the first attempt. It seems that the MR depth should be at the minimum level of MR4.5 and its duration is at least 2 years for successful TFR2.

Most studies used sustained DMR at MR4 or MR4.5 levels as eligibility criteria for more than a year or two. The duration of TKI treatment at the time of patient recruitment was also important for eligibility, which was on average around 2–3 years. The largest TFR study—the EURO-SKI—has shown that a longer duration of TKI and DMR treatment, as well as prior IFN-α therapy, correlate with a higher percentage of effective TFR [90]. Furthermore, the longer duration of TKI treatment relates to a DMR. It has been reported that the success of TFR is significantly lower in patients with stable MMR who have not achieved DMR [91]; however, this requires further research. Upon re-initiation of therapy, 90–95% of patients regain their initial molecular level or at least regain MMR. The ELN expert panel highlights that some patients have fluctuations in *BCR-ABL1* level, which sometimes improve over time without restarting therapy [49]. Although careful comparison of TFR research studies is limited by differences in eligibility criteria and definitions of DMR as well as molecular relapse, these have established standards for the safe and effective conduct of TFR studies (Table 4).

The recent ELN recommendations have underlined the importance of long-term systematic monitoring of patients after treatment is stopped due to the possibility of late relapses.

Patients’ motivation and attitude towards TFR seem to be very important, especially for women wishing to become pregnant and younger patients. Additionally, in such patients, the option of switching to 2G TKI therapy may be considered for achieving a faster and deeper molecular response, provided the DMR has been achieved so far. However, there are also patients who do not want to discontinue treatment despite meeting the inclusion criteria and such patients should not be included in the TFR trials.

## 7. Immune System-Specific Markers in CML

The clinical course of CML involves not only the dysregulation of tyrosine kinase function, but also manifests as a deep dysfunctional immune response against tumor cells expressing the fusion gene *BCR-ABL1*. In CML, like in other malignancies, the immune response against cancer is impaired, resulting in immune escape of the malignant cells, which supports cancer development. Active immunotherapy represents a tempting approach directing not only the immune response against leukemic cells but also restoring immune function. Searching for the most meaningful immunogenic leukemia-associated antigens as novel targets for immunotherapy and assessment of CML-specific immune response in CML patients could help to characterize the role of TKIs in promoting a long-term response. Recent studies proved the efficient induction of specific cytotoxic T lymphocytes (CTLs) directed against leukemia-associated antigens: *BCR-ABL1*, the receptor for hyaluronan-mediated motility (RHAMM), Wilms Tumor-1 (WT-1), proteinase 3 (PR3), preferentially expressed antigen in melanoma (PRAME), M-phase phosphoprotein (MPP11), Aurora A kinase (AURKA), and human chromosome X open reading frame 48 (CXorf48) in CML [92,93,94,95,96,97,98,99,100]. Other antigens like renal cell carcinoma-associated antigen (NEWREN60) and sperm-associated antigen 9 (SPAG9) demonstrated restricted and upregulated mRNA expression in CML, although they have not been functionally investigated yet [92,101,102]. Moreover, patients with CXorf48-specific CTL had a higher rate of successful TFR after imatinib than those without [103]. All of these molecules represent novel promising targets for peptide-based immunotherapy and they could act as markers for assessment immune responses in CML patients with a deep molecular response.

Regulatory T cells (Treg) are a group of immunosuppressive T cells that play a crucial role in self-tolerance and immune response against tumor cells. Characteristic features of Tregs cells are their anergic state, the ability of active inhibition of CD4^+^CD25^-^ T cells, CD8^+^ T cells, dendritic cells (DC), natural killer (NK) cells, natural killer T (NKT) cells, and B cells in a cell-to-cell contact and dose-dependent manner. It may facilitate tumor cell proliferation and it is one of the mechanisms of tumor cells’ escape from immune surveillance. Increased proportions or functional activities of Treg are found in patients with solid or hematological malignancies, thereby providing strong evidence that Treg may play a critical role in suppressing an effective antitumor immune response [104]. Bachy et al. [105] have shown that Treg are significantly increased in CML patients with intermediate- or high-risk Sokal scores when compared with low-risk patients. Furthermore, Zahran et al. [106] found an increased percentage of Tregs in CML patients in comparison to healthy controls. They also found that Treg numbers were lower in patients with chronic phase CML versus accelerated and blast phases and were significantly decreased in patients with CMR when compared to those patients without CMR. Similar reports have been given by Hus et al. [107], who reported that the percentage of CD4^+^CD25^high^FoxP3^+^ cells was higher in untreated CML patients compared to healthy controls. Additionally, Rojas et al. [108] demonstrated an increased frequency of Treg in patients with high levels of *BCR-ABL1* transcripts than in cases with low levels of *BCR-ABL1* gene transcripts or healthy donors. In another study, Nadal et al. [109] observed that Tregs cells are increased in patients after alloSCT when compared with healthy controls (median 1.5 vs. 0.78%, *p* = 0.004). Interestingly, the analysis of the newly diagnosed CML patients showed that not only the Tregs frequency was markedly reduced compared to patients after alloSCT (median 0.27 vs. 1.5%, *p* = 0.0003), but also with respect to healthy volunteers (median 0.27 vs. 0.87%, *p* = 0.03). Moreover, they found significantly higher Treg cells numbers in CML patients that relapse after alloSCT, suggesting that these cells may be detrimental to the graft vs. leukemia effect of allogeneic stem cell transplantation. Several studies have reported a complex influence of TKI, such as imatinib, on the improvement of the immune system function. Such therapy on the one hand has a positive effect by enhancing anti-tumor function of CD4^+^ T cells and dendritic cells, but on the other hand might reduce the proliferation and activity of Treg cells. Data from in vitro and in vivo studies on mice models reported a significant downregulation of proliferation, activation, FOXP3 expression, and an impaired immunosuppressive function of CD4^+^CD25^high^ Tregs by imatinib in a dose-dependent manner [110]. Lu et al. [111] showed that treatment with TKI reduces the percentage of T, Treg, CD4^+^, and CD8^+^ T cells to a different extent depending on the TKI used in CML patients compared to healthy controls. The decrease in the amount of Treg gradually deepened with the duration of the treatment. This study also demonstrated inhibition of Treg function, including proliferation, suppression, and expression of IL-4, IL-10, TGF-β cytokines, and FOXP3, GITR, and CTLA-4 molecules in the imatinib and dasatinib treatment groups, which was not observed in patients treated with nilotinib. A study by Dai et al. [112] indicated that in CML patients treated with dasatinib, it has the strongest effect on increasing the Th1 percentage compared to imatinib and nilotinib treatment, while reducing the Treg lymphocyte population, thus increasing the chance of successfully achieving MMR and MR4.5. Important observations were made by Alves et al. [113], who proved that CML patients treated with 2G TKI had a lower percentage of CD4^+^ Treg and granulocytic myeloid-derived suppressor cells (Gr-MDSC) compared to patients receiving imatinib (median CD4^+^ Treg 3.63 vs. 6.18%, *p* = 0.005 and Gr-MDSC 4.2 vs. 8.2%, *p* = 0.003), but higher levels of PD-1-co-expressing CD4^+^ cells (1.92 vs. 1.0%; *p* = 0.001). 

CML patients with low levels of CD62L on T lymphocytes and high levels of soluble CD62L in plasma induce a pro-inflammatory leukemic environment, while nilotinib increases CD62L expression on T lymphocytes and thus improves antitumor immunity [114]. In turn, dasatinib significantly supports the anti-leukemic response by inducing the expansion of large granular lymphocytes (LGL), such as NK cells and CD8^+^ T cells. Due to the generation of a strong cytotoxic memory response, LGLs can effectively destroy cancer cells even after cessation of treatment [115,116].

Another subpopulation of the blood cells that might play a crucial role in immune system restoration after TKI treatment is natural killer (NK) cells. NK cells are effector lymphocytes of the innate immune system that control several types of tumors and microbial infections by limiting their spread and subsequent tissue damage. They are defined as CD3^−^CD56^+^ lymphocytes, distinguished as CD56^bright^ and CD56^dim^ subsets. Activated NK cells are in a position to directly or indirectly exert their antitumor activity to control tumor growth and prevent rapid dissemination of metastatic tumors by immune surveillance mechanisms [117]. The inhibitory killer cell immunoglobulin-like receptors (KIR) and their human leukocyte antigen (HLA) ligands play a crucial role in the antitumor activity of NK cells, and their mismatch in the context of the major histocompatibility complex (MHC) may trigger alloreactivity of NK cells [118]. Moreover, NK cell alloreactivity is of great importance in controlling a graft-versus-host (GVH). Interestingly, the genotype of the KIR receptor is also important as the presence of KIR3DS1/KIR3DL1/HLA-Bw4 was shown to be significantly associated with relapse after the TFR test. Cumulative TFR is significantly higher in patients homozygous for the KIR A haplotype. Previously reported results indicate that NK cells of newly diagnosed CML patients are in reduced number or proportion among lymphocytes and have limited cytolytic capacity at the diagnosis of CML [99,119,120]. A key role in the cytotoxicity of NK cells is played by receptors activating mainly NKG2-D type II integral membrane protein (NKG2D) and their NKG2DL ligands, including MICA, MICB, ULBP1, and ULBP2, which are expressed on CML blasts. Although there is a lack of specific prognostic factors that could determine which patients could discontinue the therapy, there is increasing evidence suggesting that NK cells are important in the control of leukemic growth. In animal experiments, it has been shown that NK cells after implanting them in the irradiated bone marrow of the recipient mice can control leukemic cells [121]. In CML patients, increased NK cell counts seem to correlate with successful imatinib discontinuation. Boissel et al. [122] demonstrated that at the time of diagnosis, patients with CML had abnormally high levels of soluble forms of MIC (sMIC) in the serum and poor NKG2D expression on NK and CD8^+^ T lymphocytes, which were restored by imatinib therapy. Ohyashiki et al. [123] reported that CML patients who sustain a CMR after imatinib discontinuation have higher levels of NK cells than normal subjects or CMR patients under imatinib therapy do. In other study, Mizoguchi et al. [124] observed a significantly higher percentage of effector populations of NK cells after stopping imatinib in CMR groups than in the fluctuating CMR and control groups. The elevated levels of these effector NK cells were sustained for more than 3 years after imatinib discontinuation. In addition, imatinib treatment activates NK to the intensified release of IFN-γ in gastrointestinal stromal tumor patients [125]. The most recent studies reported that after imatinib discontinuation, the NK cell level significantly increased in non-relapsing CML patients and remained higher in comparison with relapsing patients [126]. Moreover, Ilander et al. [127] suggested that non-relapse patients demonstrated a higher than the median CD56^dim^ NK cell subset percentage at the time of imatinib withdrawal and had a better probability of staying in remission. NK cells have been an attractive tool for several years in cancer immunotherapy, an important approach of which is to block checkpoints, such as interactions programmed death-1 (PD-1)/programmed death ligand-1 (PDL-1), IL-1, and JAK/STAT pathways [120].

These results suggest that the immunological activation status of NK cells contributes to DMR maintenance. Higher activation levels of effector NK cells in CML patients treated with TKI might reflect minimization of *BCR-ABL1* transcript levels and therefore could serve as additive information when determining imatinib discontinuation. The function and genetic determinants of the NK-cell expansion need further study, but it is tempting to speculate that in the future, successful CML therapy should aim to increase the number and activity of NK cells. Recently, an additional NK subpopulation of interesting T-cell properties (NKT) that might play a role in the anti-leukemia immune response was defined. Invariant NKT cells (iNKT) have been reported to be dysfunctional, but their function improved in patients who achieved CCyR with IFN-α or TKI treatment [128]. There is no data about the function of NKT cells in patients who stopped TKI therapy.

TKIs affect a broad spectrum of kinases, thereby exhibiting a negative impact on the proliferation and activation of multiple different blood cell types, including T cells [129]. Recent data suggest that inhibition of dendritic cell (DC) generation in imatinib-treated patients contributes to a reduction of CTL levels, due to ineffective peptide priming. Moreover, imatinib treatment seems to directly inhibit T cell receptor-mediated proliferation and activation results in delayed-type hypersensitivity in CML patients. In turn, long-term imatinib treatment might also induce the immune response of CD4^+^ T cells with the generation of CD4^+^ and CD8^+^ BCR-ABL1 specific effector-memory T cells [130,131]. All of these studies emphasized the important role of imatinib in the modulation of anti-leukemic response in CML patients. Plasmacytoid dendritic cells (pDCs) can induce immunosuppressive Treg lymphocytes and reduce the functions of NK cells and T lymphocytes [132]. Inselmann et al. noted that a higher CML-pDC count at diagnosis was associated with a poorer severity of the molecular response to nilotinib unless nilotinib therapy was combined with IFN (CML-V study), which strongly decreased the total number of circulating pDC, including CD86^+^ pDC [133]. Schütz [134] suggested that low levels of CD86^+^ on pDC may be a predictor of TFR as it increases RFS for patients with <95 CD86^+^ pDC compared to patients with> 95 CD86^+^ pDC per 105 lymphocytes (70 vs. 30.1%; *p* < 0.0001). Moreover, high levels of CD86^+^ pDC correlated with depletion of the leukemia-specific CD8^+^ T cell populations. In tumor immunotherapy using DC, cross-presentation of a sufficient amount of tumor antigens to effectively stimulate a CTL response may be of key importance [135]. This approach was used by Yang [136], who, in order to induce the cross-presentation of exogenous antigens derived from the BCR-ABL1 oncoprotein by DCs, was fused with cytoplasmic transduction peptides. In this study, the induced CTL showed a stronger ability to kill CML cells.

To date, the only curative therapy procedure for TKI non-responder CML patients remains alloHSCT. However, there are multiple pieces of evidence for the efficiency of immunotherapy treatment in CML, burdened with less mortality and morbidity. Previous studies compared the results of allogeneic bone marrow transfer with and without T cell depletion before the procedure. They found that the disposal of T cells from the graft results in a higher relapse rate in CML patients, suggesting an extremely important role of the T cell population in the eradication of leukemia cells [137]. Moreover, a clinical trial demonstrated that 40% of patients who discontinue imatinib did not show relapse symptoms that might manifest a T cell-mediated immune response with long-term memory [68].

Programmed death 1 (PD-1) is a member of CD28 co-stimulatory molecules, expressed on activated T and B cells, NK, and myeloid cells. PD-1 controls peripheral tolerance through binding to corresponding ligands PD-L1 and PD-L2, and inhibits proliferation, cytokine production, and cytotoxic function of effector cells. Recent studies demonstrated that elevated PD-1 expression on specific CTL directed against CML cells is related to loss of CTL lytic activity after co-stimulation with PD-L1-positive CML cells [138]. There was also evidence that PD-1 could be upregulated in CD4^+^ cells in CML patients [139]. Mumprecht et al. [140] proved the expression of PD-1 on CML-specific CTLs and the expression of PD-1 ligand (PD-L1) on CML cells in a mouse model, while extending the study to CML patients, who demonstrated increased PD-1 expression on their CD8^+^ T cells. These results suggest that PD-1 might contribute to maintaining immunity compromise during the chronic phase of the disease. Moreover, increased expression of PD-1 in CD8^+^ cells might be associated with disease progression and represents the mechanism of tumor escape from immunosurveillance [140]. Interestingly, Riether et al. [141] showed that PD-1 blockade in combination with immunotherapy could eradicate leukemic stem cells and decrease tumor burden in CML mice. However, there are no available data about alterations in PD-1 expression after discontinued imatinib treatment in CML patients. Investigations of the protein changes during the remission phase or in relapse may clarify if the blockade of the PD-1 molecule in CML could carry benefits in relapse patients.

Another modulator of the immune system that might influence maintaining TFR in CML patients is an infection of cytomegalovirus (CMV). CMV is the largest member of the virus family Herpesviridae and is a ubiquitous virus that infects almost all humans at some time in their lives. Symptomatic CMV infection predominantly occurs in immunocompromised hosts, such as patients after alloSCT, whereas symptomatic infection of healthy persons is rare [142]. There are relatively many studies showing an association of CMV reactivation with a reduced number of acute myeloid leukemia (AML) relapses following allo-SCT. Behrendt et al. [143] observed a significantly higher leukemic relapse risk after alloSCT myelodysplastic syndrome patients with a negative pre-transplantation CMV donor and recipient serostatus compared with patients with a positive pre-transplantation CMV serology of donors or recipients. In another study, Emaagacli et al. [144] reported that CMV reactivation is associated with a decreased risk of disease relapse in patients with AML. Furthermore, Green et al. [145] found that CMV reactivation in the first 100 days after transplantation is associated with a modest decrease in the risk of early relapse independent of acute GvHD in patients with AML. There are also studies in the literature on the favorable ‘virus-versus-leukemia’ effect from reactivating CMV in CML transplant recipients. Ito et al. [146] showed that CMV viral reactivation as a time-dependent covariate was an independent factor associated with a reduction in relapse in CML patients after allo-SCT. CMV reactivation may contribute to the beneficial effects of graft-versus-leukemia (GVL) by stimulating the immune system. It has been shown that subclinical CMV reactivation can lead to the expansion of specific subsets of NK and T cells with both antitumor and antiviral activity due to the common pathways of the immune response [147]. Kadowaki et al. [148] observed a relationship between CMV reactivation and expansion of LGL, which include CD8 + T lymphocytes, γδT lymphocytes, and NK cells. Additional analyses showed that NK cells in CMV + seropositive patients underwent phenotypic progression that was further enhanced by TKI, including dasatinib, in this study. Thus, CMV-associated NK cell activation may help to prevent CML recurrence upon discontinuation of treatment. The protective mechanism of CMV reactivation during relapse is unknown. There is a hypothesis that CMV reactivation might induce T cell and NK cell attack on malignant cells due to latent CMV virus presence or the activation of CTL affecting leukemic cells. NK cells play a crucial role in curtailing CMV and other viral infections in immunocompetent individuals through the expression of a series of activating receptors (NKG2D, DNAM-1, NKp46) that allow them to recognize and eliminate CMV and other virus-infected cells [142]. In in vitro experiments using IL-2-activated human NK cells, Iversen et al. [149] demonstrated that NK cells can inhibit CMV replication in CMV-infected fibroblasts by inducing IFN-beta release from infected fibroblasts. Kheav et al. [150] reported that a higher percentage of NKG2C+ NK cells is associated with a lower risk of acute CMV infection in patients undergoing solid organ transplantation or HSCT. Foley et al. [151] demonstrated increased populations of IFN-γ-producing NKG2C + NKG2A − NK cells in HSCT recipients after CMV viremia. TKI treatment induces a strong immune response that persists after treatment discontinuation, and which may also be antiviral. The research of Vigón et al. [152] demonstrated that seropositive for CMV, but without CMV viremia undergoing the TFR trial previously, stimulated with CMV peptides had an 8-fold increased expression of TCRγδ and produced over 18-fold more IFNγ from CD3^+^CD8^+^ T cells similar to CMV reactivated kidney transplant patients. However, the CML patients undergoing TFR did not reactivate CMV. Moreover, it was proved that γδT cells were able to recognize both CMV-infected cells and primary leukemic blasts; therefore, it has been proposed that γδ T cells recognizing CMV peptides cross-react with leukemia cells [153]. This may indicate that the presence of CMV along with leukemia cells and treatment with TKI may elicit a strong immune response against leukemia cells as well as virally infected cells. Therefore, it may be suggested that CMV together with TKIs modulate the immune system, so that immune control over the clone of malignant CML cells may be restored. The importance of CMV status in the donor and recipient is important to the success of the transplant. There is increasing evidence suggesting that stem cell transplant recipients receiving CMV seropositive donors show improved survival, event-free survival, reduced transplant-related mortality, or reduced risk of relapse [143,154]. However, studies emphasize that in CML patients, T cell depletion offsets the beneficial effects of donor status, suggesting that ultimately it is mediated by donor immunity. Nevertheless, it is difficult to clearly define to what extent CMV reactivation is a substitute for immunological factors that directly contribute to relapse.

Moreover, the immunomodulatory effect of TKIs promotes a strong cytotoxic response, which may be a breakthrough in the fight against HIV-1 infection. Salgado et al. [155] suggested that the primary antiviral mechanism against HIV-1 was based on inhibition of SAMHD1 phosphorylation in CD4^+^ T cells, which prevented ex vivo HIV-1 infection in CML patients treated with dasatinib. However, extending their research to the group of CML patients in TFR, they noticed that despite the unblocking of the SAMHD1 phosphorylation pathway due to the withdrawal of TKI, the frequency of proviral integration was more than 12-fold reduced [152]. This creates an opportunity for the transient use of TKIs in HIV-infected patients to modulate the immune response.

Studies on the immune status of CML patients in TFR were conducted by Hughes et al. [156], who showed that CML patients at diagnosis had impaired NK cell and CTL effector function that was restored by treatment when patients achieved MMR and DMR. More importantly, sufficient effector responses were maintained upon discontinuation of treatment in imatinib-treated patients with persistent DMR. It turns out that in the restoration of immune control over the leukemic clone, there is a depth of the molecular response achieved, because the cytolytic function of NK cells, and the decrease in Mo-MDSC (monocytic myeloid-derived suppressor cells) and PD-1 expression on CD4^+^ and CD8^+^ T cells was completely restored only in MR4.5 [156]. Maintaining a sustained anti-leukemic response and a successful TFR is accompanied by a decrease in the level of Treg [80] and an increase in the level of NK cells [127]. Immunological factors relevant to the success of TFR are summarized in Table 5.

## 8. TKI Withdrawal Syndrome

The possibility to recover the proper function of the immune system during treatment with TKI and the restoration of immune control over a clone of malignant CML cells is most probably critical to achieve a long-term TFR. The importance of immunocompetent cells involved in the course of TKI discontinuation is also underlined by the presence of symptoms occurring in some patients with so-called “withdrawal syndrome” (WS). The etiology of this phenomenon remains elusive, but the hypothesis on its development involves cytokine release as a result of tyrosine kinase unblocking. TKI WS affects approximately 25–30% of patients after discontinuation of treatment [157,158] (Table 6). It usually presents as diffuse musculoskeletal pain from mild intensity relieved with common painkillers or non-steroidal anti-inflammatory drugs to moderate intensity requiring the use of corticosteroids [159]. The arms, hips, and extremities are the areas that are affected by the withdrawal syndrome [62,159,160]. In research on the risk of TKI withdrawal syndrome, Berger et al. [159] obtained a significantly higher incidence of WS after discontinuation of 2G TKI than with imatinib by analyzing a cohort of 427 patients from EURO-SKI and STIM2 studies. The pain may be related to the biological effects of inhibition of the c-Kit tyrosine kinase receptor through changes in sensitivity to thermal pain [161]. TKI WS may be caused by inflammation after TKI withdrawal due to the immunomodulatory effect of TKIs. However, most patients did not have elevated proinflammatory markers, including CRP protein, or the presence of autoimmune markers, which requires further studies [160]. Interestingly, a study in South Korea showed that itching is a manifestation of TKI withdrawal syndrome [162], which was not found in the analysis of patients in EURO-SKI and STIM2, which may highlight the differences in WS symptoms in patients from different ethnic groups [159,163].

## 9. Summary

According to the latest ELN recommendation from March 2020, TFR should be considered as a new goal for the treatment of CML, which has become a challenge on the way to curing CML. This approach is primarily aimed at improving the quality of life of CML patients and avoiding long-term toxicity of TKIs. The effectiveness of TFR tests reaches almost half of CML patients undertaking the TFR test. The maturing individualized approach to the patient allows for careful monitoring of the patient. Discontinuation of treatment should be specifically justified in patients with CML with stable DMR and in patients with a high priority for TFR, e.g., women wishing to become pregnant and younger patients, depending on whether the duration of treatment with both TKI and DMR is long enough. The immune mechanism that might be responsible for sustained TFR has not yet been clearly defined.

## Figures and Tables

**Table 1:** First- and second-line-generation TKIs.

TKI	Specificity of TKI	MMR	DMR	Changes to Another TKI	OS, PFS	Side Effect
Imatinib (IM)	1G TKI the first choice for the treatment of CML	20–59%/1 years 60–80%/5 years	MR4 or deeper: 35–68%/5 years	37% ^a^ and 50% ^b^/5 years 26.5% ^c^/10 years	OS: 90–95%/5 years 82–85%/10 years PFS: 80–90%/5 years 6% leukemia-related death rate ^c,d^	no life-threatening complications ^c,d^ early fluid retention, gastrointestinal symptoms, muscle cramps, joint pain, skin rash, fatigue ^e^
Nilotinib (NIL)	2G TKI active against *BCR-ABL1* mutants: V299L, F317L/V/I/C, T315A	77% ^b^/5 years 82.6% ^f^/10 years 98% ^g^/10 years	MR4: 66%/5 years 73%/10 years 76% ^g^/10 years MR4.5: 54%/5 years 64%/10 years	40%/10 years	OS: 94%/5 years 87.6%/10 years 94% ^g^/10 years	cardiovascular events ^h^ pancreatitis ^b,f,g^
Dasatinib (DASA)	2G TKI active against *BCR-ABL1* mutants: Y253H, E255V/K, F359V/I/C	46% ^a^/1 year 76% ^a^/5 years	MR4.5: 42%/5 years	39%/5 years	OS: 91%/5 years PFS: 86%/5 years	pleuro-pulmonary toxicity recurrent pleural effusions rarely pulmonary arterial hypertension ^a^
Bosutinib ^i^ (BOS)	2G TKI active against *BCR-ABL1* mutants: Y253H, E255V/K, F359V/I/C, F317L/V/I/C, T315A	47% ^j^/1year	NR	NR	NR	transient diarrhea transient elevations of transaminases ^k^

^a^ [21] ^b^ [22] ^c^ [9] ^d^ [23] ^e^ [24,25] ^f^ [26] ^g^ [27] ^h^ in about 20% of patients over a 10-year period vs. 5% for IM [22,26,27] ^i^ data on clinical mutation-related resistance to bosutinib are limited ^j^ [28] ^k^ [29].

**Table 2:** Scoring molecular response.

Type of Response	*BCR-ABL1* Levels ^a^	Reduction in *BCR-ABL1* Transcript Levels ^b^	Sum of Reference Gene Transcripts ^c^
CCyR ^d^	≤1%	≥2 log	≥10,000 *ABL1* ^i^ or 24,000 *GUSB* ^j^
MMR or MR3 ^e^	≤0.1%	≥3 log	≥10,000 *ABL1* or 24,000 *GUSB*
MR4 ^f^	≤0.01%	≥4 log	≥10,000 *ABL1* or 24,000 *GUSB*
MR4.5 ^g^	≤0.0032%	≥4.5 log	≥32,000 *ABL1* or 77,000 *GUSB*
MR5 ^h^	≤0.001%	≥5 log	≥100,000 *ABL1* or 240,000 *GUSB*

^a^ on the International Scale (IS) [18] ^b^ from IRIS baseline representing the median value of BCR-ABL1/BCR present at diagnosis [44] ^c^ numbers of ABL1 transcripts in the same volume of cDNA used to test for BCR-ABL1 ^d^ CCyR—complete cytogenetic remission, ^e^ MMR/MR3—major molecular response, ^f^ MR4—undetectable disease in cDNA with >10,000 ABL1 transcripts, ^g^ MR4,5—undetectable disease in cDNA with >32,000 ABL1 transcripts, ^h^ MR5—undetectable disease in cDNA with >100,000 ABL1 transcripts, ^i^ minimal sensitivity for accurate quantification [49], ^j^ GUSB—beta glucuronidase.

**Table 3:** Clinical trials of TFR.

Study	Pts	Treatment before TFR	DMR	TFR	Criteria for Molecular Relapse	Percentage of pts with Relapse **
**Studies on IMATINIB**						
STIM1 [67] updated at ESH 2019, [68]	100	IM (1st line) ≥ 3 years	UMRD * ≥ 2 years	43% after 6 months 41% after 1 year 40% after 1.5 years 38% after 5 years 38% after 7 years 37% after 10 years	loss of UMRD on 2 consecutive tests or MMR on 1 test	61%
TWISTER [69,70]	40	IM (1st line) ≥ 3 years	UMRD ≥ 2 years	47% after 2 years 45% after 3.5 years 45% after 8.5 years	loss of UMRD on 2 consecutive tests or MMR on 1 test	55%
A-STIM [64]	80	IM (1st line) ≥ 3 years	UMRD ≥ 2 years	64% after 1 year 64% after 2 years 61% after 3 years	loss of MMR	36% ^##^
ISAV [71]	112	IM (1st line) ≥ 2 years	UMRD ≥ 1.5 years	48% after 3 years 46% after 6.5 years	loss of UMRD on 2 consecutive tests or MMR on 1 test	52%
KID [72]	126	IM (1st line) ≥ 3 years	UMRD ≥ 2 years	62% after 1 year 59% after 2 years	loss of MMR on 2 consecutive tests	44%
TRAD [73]	75	IM (1st line) ≥ 3 years DASA (2nd line)	MR4.5 ≥ 2 years	65% at 6 months 57.5% after 1 year	loss of MR4 on 2 consecutive tests or MMR on 1 test	31% ^###^
**Studies on NILOTINIB**						
STAT2 [74]	78	IM/NIL (1stline) NIL (2nd line) ≥ 2 years	MR4.5 ≥ 2 year	68% after 1 year 63% after 3 years	loss of UMRD on 2 consecutive tests or MMR on 1 test	37%
ENESTFreedom [75] updated EHA 2018, [76]	190	NIL (1st or 2nd line) ≥ 2 years	MR4.5 > 1 year	63% after 6 months 52% after 1 year 49% after 2 years 47% after 3 years	loss of MMR	48%
ENESTop [26,77,78]	126	IM (1st line) NIL (2nd line) ≥ 3 years	MR4.5 > 1 year	58% after 1 year 46% after 4 years 43% after 5 years	loss of MR4 on 2 consecutive tests or MMR on 1 test	47%
NILst [79]	87	IM/NILO (1st line) NILO (2nd line) ≥ 2 years	MR4.5 ≥ 2 years	61% at 1 year unchanged after 3 years	loss of MR4.5 on 2 consecutive tests	39%
**Studies on DASATINIB**						
DADI [80,81]	63	IM (1st line) DASA (2nd line or subsequent) ≥ 2 years	[*BCR-ABL1* ≤0.0069] > 1 year	49% after 6 months 48% after 1 year 44% after 3 years	*BCR-ABL1* > 0.0069%IS loss of MR4	56%
first-line DADI trial [82]	58	DASA (1st line) ≥ 2 years	[*BCR-ABL1* ≤0.0069] > 1 year	55% after 6 months unchanged after 1 year	*BCR-ABL1* > 0.0069%IS loss of MMR	45%
D-STOP [83]	54	IM (1st line) DASA (1st or 2nd line) ≥ 2 years	UMRD MR4 > 2 years	69% after 6 mts 63% after 1 year 57% after 2 years	loss of MR4 on 2 consecutive tests	43%
DASFREE [84]	84	IM (1st line) DASA (1st line or subsequent) ≥ 2 years	MR4.5 ≥ 1 year ***	48% after 1 year 46% after 2 years	loss of MMR	55%
**Studies on IMATINIB, NILOTINIB and DASATINIB**						
STOP 2G-TKI (pilot) [85]	60	(IM (1stline)) NIL/DASA (1st, 2nd or 3rd line) ≥ 3 years	UMRD MR4.5 ≥ 2 years	63% after 1 year 54% after 4 years	loss of MMR	43%
EURO-SKI [86]	755	IM/DASA/NIL (1st or 2nd line) ≥ 3 years	MR4 ≥ 1 year	61% after 6 months 50% after 2 years 47% after 3 years	loss of MMR	49%
DESTINY [87]	157 ^@^	IM/DASA/NIL (1st line) ≥ 3 years	MR4/MMR ≥ 1 year	64% after 3 years ^@@^	loss of MMR on 2 consecutive tests	41% ^@@@^
**2nd TFR attempt (TFR2)**						
RE-STIM [88] udated at EHA 2019	106	re-attempted TKI discontinuation after a first unsuccessful attempt	regained MR4.5 ^a^	48% after 1 year 42% after 2 years 35% after 3 years 33% after 4 years	loss of MMR	64% ^#^
TRAD2 [89]	25	(1) IM discontinuation phase; (2) DASA rechallenge phase; (3) DASA discontinuation phase.	MR4 > 1 year	21.5 ± 8.5% after 6 months	loss of MR4 on 2 consecutive tests or MMR on 1 test	84%

* undetectable molecular residual disease; ≥4.5 log or ≥5 log sensitivity; ** in last year of follow-up; *** the duration of MR4.5 before treatment discontinuation is only available for 74 of 84 patients due to a retrospective assessment; ^#^ [88] ^##^ 56% loss of CMR ^###^ 21/67 pts ^@^ 39 pts with MMR and 118 pts with M4 ^@@^ 71% pts with MR4 and 41% pts with MMR ^@@@^ 29% pts with MR4 and 77% pts with MMR ^a^ after any TKI resumption.

**Table 4:** Requirements for tyrosine kinase inhibitor discontinuation.

Requirements for tfr-Recommendations ELN		
Mandatory	Minimal	Optimal
CML in first CP motivated patient rapid access to quality-controlled PCR tests using the IS	TKIs first-line or second-line (if switching to the second line is due to intolerance), e13a2 or e14a2 transcripts TKI therapy > 5 years or 2G TKI > 4 years, MR4 or better > 2 years no prior treatment failure	TKI therapy > 5 years MR4 > 3 years MR4.5 > 2 years

**Table 5:** Immune system factors critical to the success of TFR.

Immunological Factors Supporting tfr	Modulation
decreased proportions and functional activities of regulatory T cells (Treg)	↓
decreased expression of IL-4, IL-10, TGF-β cytokines and FOXP3, GITR, CTLA-4 molecules	↓
enhancing anti-tumor function of CD4^+^ and CD8^+^ T cells	↑
induction of specific cytotoxic T lymphocytes (CTL)	↑
increased expression of TCRγδ and produced IFNγ from CD8^+^ T cells	↑
increased expression of CD62L on T cells	↑
generation of CD4+ and CD8+ BCR-ABL1 specific effector-memory T cells	↑
expansion of large granular lymphocytes (LGL)-NK cells and CD8+ T cells	↑
increased proportions of CD56^dim^ NK cell subset	↑
enhancing cytotoxic activities of NK and NKT cells	↑
increased expression of NKG2D, DNAM-1, NKp46 and homozygous for the KIR-A haplotype on NK cells	↑
improved effective peptide presentation function of dendritic cells (DC)	↑
decreased expression of CD86^+^ on plasmacytoid dendritic cells (pDC)	↓
decreased proportions of monocytic and granulocytic myeloid-derived suppressor cells (Mo-MDSC and Gr-MDSC)	↓
decreased PD-1 expression on CD4^+^, CD8^+^, NK and DC cells	↓

**Table 6:** TKI withdrawal syndrome.

Study	Treatment	Percentage of Patients with WS
EURO-SKI [86]	IM/NIL/DASA	30.7%
KID [162,72]	IM	30%
JALSG-STIM213 [164]	IM	14.7%
ISAV [71,165]	IM	21.2%
A-STIM [64]	IM	5%
STIM2 [159]	IM	21%
ENESTfreedom [76]	NIL	24.7%
ENESTop [78]	NIL	48%
STAT2 [74]	NIL	14.1%
DASFREE [84]	DASA	11%

## Data Availability

Not applicable.
